# Detection of new pioneer transcription factors as cell-type specific nucleosome binders

**DOI:** 10.1101/2023.05.10.540098

**Published:** 2023-05-12

**Authors:** Yunhui Peng, Wei Song, Vladimir B. Teif, Ivan Ovcharenko, David Landsman, Anna R. Panchenko

**Affiliations:** 1 current address: Institute of Biophysics and Department of Physics, Central China Normal University, Wuhan 430079, China; 2 National Library of Medicine, National Institutes of Health, Bethesda, MD, USA; 3 School of Life Sciences, University of Essex, Wivenhoe Park, Colchester, CO4 3SQ, UK; 4 Department of Pathology and Molecular Medicine, Queen’s University, ON, Canada; 5 Department of Biology and Molecular Sciences, Queen’s University, ON, Canada; 6 School of Computing, Queen’s University, ON, Canada; 7 Ontario Institute of Cancer Research, Toronto, ON, Canada

## Abstract

Wrapping of DNA into nucleosomes restricts DNA accessibility and the recognition of binding motifs by transcription factors. A certain class of transcription factors, so-called pioneer transcription factors, can specifically recognize their binding sites on nucleosomal DNA, initiate local chromatin opening and facilitate the binding of co-factors in a cell-type-specific manner. For the vast majority of human pioneer transcription factors, the locations of their binding sites, mechanisms of binding and regulation remain unknown. We have developed a computational method to predict the cell-type-specific ability of transcription factors to bind nucleosomes by integrating ChIP-seq, MNaseq-seq and DNase-seq data with the details of nucleosome structure. We have achieved classification accuracy with AUC=0.94 in discriminating pioneer factors from canonical transcription factors and predicted 32 potential pioneer transcription factors as nucleosome binders in embryonic cell differentiation. Lastly, we systemically analyzed the interaction modes between various pioneer factors and detected several clusters of distinctive binding sites on nucleosomal DNA.

## Introduction

In eukaryotic cell, DNA is packaged in the form of chromatin but at the same time, it should be dynamically accessed during transcription and replication processes at high spatiotemporal precision^1^. Open chromatin is thought to comprise actively transcribed genes and compact chromatin contains repressed genes. However, most recent observations point to a limited association between DNA accessibility, chromatin compaction and gene transcription at the global genomic scale. Indeed, rapid transcription activation may occur by relatively small changes of DNA solvent exposure, and nucleosomal array structures can be dynamically exposed without the large-scale chromatin rearrangements^2–5^. Nucleosomes represent the basic subunits of chromatin structure and comprise a histone octamer of four types of core histones of two copies each, and ~147 base pairs of DNA wrapped around them^6^. Wrapping of DNA into nucleosomes inherently restricts DNA accessibility and the recognition of binding motifs by transcription factors. Intrinsically disordered histone tails flank histone core domains and may also modulate DNA accessibility by forming transient interactions with the nucleosomal and linker DNA^7^. The control of the DNA accessibility at nucleosomal and subnucleosomal scales is of major importance in understanding how certain transcription factors can target compact chromatin to induce transcription activation or repression^8–10^.

The differentiation of cells into different lineages occurs through chromatin reprogramming involving the cooperative behavior of various transcription factors^11,12^. Although nucleosomes generally hinder the binding of transcription factors (TFs), a certain class, so-called pioneer transcription factors (PTF), can specifically recognize their binding sites on nucleosomal DNA, initiating local chromatin opening and facilitating subsequent binding of other co-factors in a cell-type specific manner^2,12^. Many recent studies have revealed the critical roles of pioneer factors in mediating the cell-type specific gene expression and establishment of cell lineage reprogramming^11–13^.

Significant efforts have been made to characterize the interaction landscape between various TFs and nucleosomes^14–17^. Using NCAP–SELEX approach, a recent study has characterized the interaction modes between nucleosomes and 220 transcription factors^14^. Another high-throughput protein microarray study of 593 human TFs systematically characterized the structural features of TFs binding with nucleosomes^18^. It has been revealed that the vast majority of TFs preferably bind free DNA instead of nucleosomal DNA, whereas certain transcription factors specifically target nucleosomes at different locations and orientations^14–16^.

Moreover, several structures of pioneer factors in complex with nucleosomes have been recently resolved^19–22^. Despite significant advances in recent experimental approaches, the interaction modes of most transcription factors with nucleosomes remain obscure and their celltype-specific pioneer activities are largely unknown. To fill this gap, the development of new computational approaches can improve our understanding of the binding properties of various TFs with nucleosomes helping to identify novel pioneer factors.

Through the advances in high-throughput sequencing techniques, a large amount of high-resolution data has been generated (ChIP–seq, ATAC-seq, Dnase-seq and MNase-seq^23^) which allows us to gain insights into the chromatin structure and details of epigenetic binding events. The rapid growth of such data has further stimulated the development of computational methods to characterize cell-type specific transcription factors^24–27^. Several machine learning models have been proposed to predict transcription factor (TF) binding sites and identify sequence context features critical for TF binding^24,26–28^. In addition, gene regulatory network-based approaches have helped to identify the key transcription factors in cell fate determination^29,30^. Despite the success of computational methods in genome-wide prediction of binding sites of canonical transcription factors, the locations of pioneer factors’ binding sites, mechanisms of binding and regulation have not been systematically explored.

**Figure 1.**
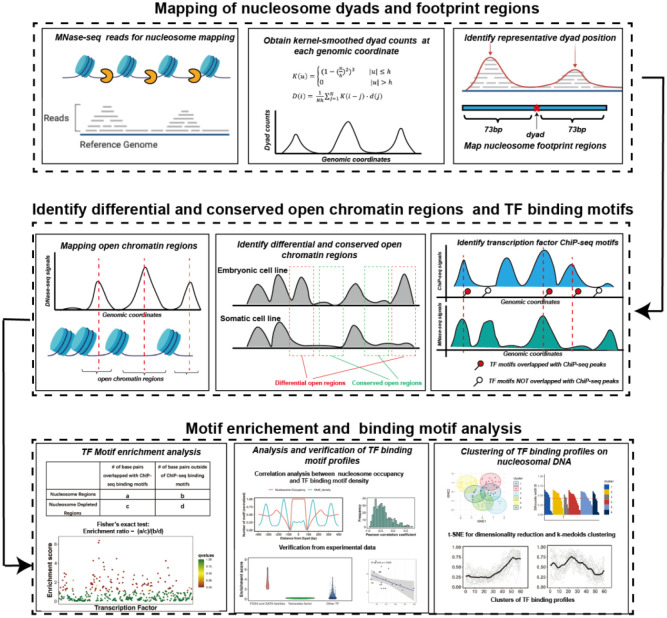
The computational framework to analyze the ability of transcription factors to bind to nucleosomes by integrating ChIP-seq, MNaseq-seq and DNase-seq data for motif enrichment and binding motif analysis.

Integrating high-resolution data on nucleosome positioning and DNA accessibility (MNase-seq, ATAC-seq and DNase-seq) with the data on DNA binding events available through ChIP-seq and other methods can reveal the interplay between transcription factor binding and nucleosome positioning, providing insights into the mechanisms of pioneer transcription factor interactions with nucleosomes and their ability to modulate chromatin accessibility^31–34^. Herein, we have developed a computational method to study the ability of transcription factors to bind nucleosomes by using ChIP-seq, MNaseq-seq and DNase-seq data from five different cell lines and 225 human transcription factors (Figure 1). First, our results point to the capability of our method to discriminate between pioneer and canonical transcription factors using experimental benchmarks. We have further predicted 32 transcription factors as potentially cell-type specific nucleosome binders with previously unknown pioneer activity in embryonic cell differentiation. Lastly, we systemically analyzed the interaction modes between various pioneer factors and nucleosomal DNA and detected several clusters of distinctive binding sites on nucleosomal DNA.

## Methods

### Genome-wide mapping of nucleosome dyads and footprint regions

The overall workflow of our computational framework is shown in Figure 1. High-coverage micrococcal nuclease sequencing data (MNase-seq) of five human cell lines (H1, HepG2, MCF-7, K562, and HeLa) from paired-end sequencing was used for nucleosome mapping (Supplementary Table 1). The raw MNase-seq data was downloaded from the NCBI Sequence Read Archive (SRA) and converted into the fastq format using SRA Toolkit^35^. Then, the downloaded fastq files were processed using the mnaseseq pipeline from nf-core (https://github.com/nf-core/mnaseseq)^36^, a recently developed bioinformatics pipeline for MNase seq data analysis. Adapter trimming of sequencing reads was performed with Trim Galore. Then, adapter-trimmed reads were mapped to the reference genome using Burrows-Wheeler Aligner (BWA)^37^. Human genome GRCh37 was used as a reference genome for reads mapping and the maximum number of mismatches in alignment was set to 4. The minimum and maximum insert sizes for filtering of mono-nucleosome paired-end reads were set to 120 and 180 base pairs. Duplicate reads were marked using Picard MarkDuplicates command (http://broadinstitute.github.io/picard/) and discarded from the analysis to avoid PCR duplication artifacts (less than 10% of reads were duplicated). Read libraries of replicates from the same experiment condition were merged into the analysis. Next, the BAM sequence alignment files were converted into BED format using bedtools v2.30.0^38^. The aligned reads with fragment sizes from 146 to 148 base pairs were selected for mapping of the representative dyad positions.

To determine the representative dyad position of nucleosomes, we implemented and modified a previously developed nucleosome mapping protocol^39^ and dyad positions were determined as midpoints of mapped MNase-seq reads^40^. Namely, we used a triweight kernel *K* as a weighting function (Equation 1) and the kernel-smoothed dyad counts D at each genomic coordinate *i* were obtained from Equation 2^41^.


(1) $$K(u)=\{\begin{array}{ll} {\left(1-{\left(\frac{u}{h}\right)}^{2}\right)}^{3} & \left|u\right|\leq h\\ 0 & \left|u\right|>h \end{array}$$

(2) $$D(i)=\frac{1}{Nh}\sum_{j=1}^{N}K(i-j)\cdot d(j)$$

Here *N* is the length of a chromosome, *d(j)* is the dyad count at genomic coordinate *j* and *D(i)* is the smoothed dyad count at genomic coordinate *i*. Small values of bandwidth *h* lead to less smoothing but more accurate estimates of dyad positions. In our case, we chose a relatively small bandwidth value *h*=15 to improve the accuracy of the mapped dyad positions.

Next, we identified the genomic locations with the local maximum values of the smoothed dyad counts using bwtool^42^ and the minimum distance between the neighboring local maxima was set to 150 base pair. Then, for every 60-bp window centered at each local maxima, one representative dyad location was determined as the dyad location with the highest number of dyad counts in this interval. Other dyad positions within the same 60-bp interval were discarded. If two or more dyad positions had the same dyad counts within the same interval, the dyad position located closest to the local maximum of the smoothed counts was selected as the representative dyad. Last, nucleosome regions (NRs) were determined as genomic regions centered at the representative dyad positions and flanked by 73 bp segments on each side.

### Genome-wide mapping of enhancers, open chromatin and nucleosome-depleted regions

To map the open chromatin and enhancer regions, we used DNase-seq, H3K27ac ChIP-seq and H3K4me1 ChIP-seq data from the following five human cell lines (H1, HepG2, MCF7, K562, and HeLa-S3, Supplementary Table 1–2). The narrow peak files were downloaded from the ENCODE and Roadmap project^43,44^. The open chromatin regions were identified as genomic regions centered at narrow peaks and flanked by 1000 bp segments on each side.

Using open chromatin regions from the DNase-seq data we identified *differential* and *conserved open chromatin* regions using the “intersect” command from the BEDTools suite^38^. Conserved open chromatin regions represent open chromatin regions that are more than 80% shared between embryonic H1 and at least one other cell line^45^. Chromatin regions with differential accessibility (“*differential open chromatin regions*”) have less than 20% overlapping between embryonic H1 and any other cell line^45^. These differential open regions may be open and active in one cell line but closed in another. Nucleosome-depleted regions (NDRs) represent genomic regions free of nucleosomes and were identified as genomic regions not covered by any mono-nucleosome fragments (120 to 180 base pairs length) in MNase-seq data in all replicate experiments. For motif enrichment analysis of transcription factors, we selected NDRs located in open chromatin regions.

### Analysis of dinucleotide patterns of nucleosomal DNA

Using identified representative nucleosome dyad positions, we examined the dinucleotide patterns of nucleosomal DNA and mapped genomic coordinates of WW/SS (where W is A or T, and S is G or C) and YY/RR (R = A or G, and Y = C or T) dinucleotides on nucleosome regions (NRs). Then, we aligned NRs by superimposing their dyad positions and computed the frequency of observed dinucleotides at each location of nucleosomal DNA (as a function of distance in base pairs from the nucleosome dyad). As a result, we observed pronounced dinucleotide patterns, known for their importance of nucleosome positioning which helped us to verify the results of nucleosome mapping (Supplementary Figure 1).

### Genome-wide mapping of transcription factor binding sites

To map the genome-wide locations of binding sites of various transcription factors (TFs), we matched the ChIP-seq data from the ENCODE project^43^ with the MNase-seq data for the same human cell lines (H1, HepG2, MCF-7, HeLa-S3 and K562) (Supplementary Table 3). We used HeLa-S3 in ChiP-seq, which is a clonal derivative of HeLA. In total, ChIP-seq data for 225 transcription factors could be matched with MNase-seq data. All available narrow peak files of ChIP-seq peaks of these transcription factors were downloaded, and files corresponding to the same transcription factor were merged for further analysis. To map binding sites from ChIP-seq narrow peaks, referred to as “ChiP-seq motif”, we downloaded position frequency matrices (PFMs) for each TF from the JASPAR CORE database^46^. Then we applied FIMO program^47^ to scan the DNA sequences within ChIP-seq narrow peaks using each PFM and identified motifs with P-values less than 10^−4^ for further analysis.

Next, we calculated the transcription factor binding motif profiles or briefly *“motif profiles”*. To do that, we first mapped ChIP-seq motifs for a given TF to the closest dyad position and aligned all dyad positions and corresponding ChIP-seq motifs (Supplementary Figure 2). For each TF motif, we considered sequences in both strands of DNA. (Supplementary Figure 2). Then, we counted the number of TF motifs (motif density) mapped at each location of nucleosomal DNA and flanking DNA regions (+/− 400 bp around the dyad region) as a function of distance in base pairs from the nucleosome dyad.

### Transcription factor motif enrichment analysis

Pioneering transcription factors (PTFs) can engage nucleosomal DNA, while truly canonical TFs cannot bind to nucleosomes. To predict pioneer factors, we calculated the binding motif enrichment of different TFs on nucleosomal regions (NRs) compared to nucleosome-depleted regions (NDRs). Namely, we counted a number of base pairs of ChIP-seq motifs overlapped with the NRs and NDRs regions and constructed the following contingency table (Supplementary Table 4). We then calculated the enrichment score for each transcription factor (Equation 4) and applied Fisher’s exact test to evaluate the significance of TF motif enrichment on NRs compared with NDRs. We further collected the data of expression levels of various TFs from the NIH roadmap epigenomics program (https://egg2.wustl.edu/roadmap/data/byDataType/rna/expression/) and identified TFs highly expressed in each cell line (RPKM value >= 10). The motif enrichment score was calculated as shown in Supplementary Table 4:

(4) Enrichment score (Odds ratio)=(a/c)/(b/d)

Where *a* and *c* are the number of base pairs overlapped with ChIP-seq binding motifs on NR and NDR respectively. Counts *b* and *d* correspond to the number of base pairs outside of ChIP-seq binding motifs on NR and NDR respectively.

Receiver operating curves (ROC) analyses were further performed to evaluate the power of enrichment scores in discriminating pioneer factors from other transcription factors. Sixteen known pioneer factors from the literature (which were included in our list of 225 TFs) were used as positives (Supplementary Table 7) and other transcription factors were considered as negatives for this test. In this study, we used two sets of NRs and NDRs for the enrichment analysis. One set corresponded to NRs located in differential open chromatin regions and NDRs located in conserved open chromatin regions. The second set included all NRs and NDRs.

### Clustering of transcription factor binding motif profiles

To identify the prevalent interaction modes of various TFs with nucleosomes, we have performed clustering of TF binding motif profiles. Prior to clustering, we filtered out potentially low-quality binding profiles using the following criteria. First, we removed underrepresented TFs with the total genome-wide cumulative sum of ChiP-seq motifs on nucleosome regions of less than 500 base pairs. Second, due to the two-fold symmetry of DNA in nucleosome structures, TF motifs on DNA complementary plus and minus strands should be structurally superimposed if the nucleosome structure is rotated by 180 degrees. Since we are analyzing a very large number of binding motifs on both DNA strands, binding motif profiles should be symmetrical with respect to the nucleosome dyad. Therefore, we calculated the Pearson correlation coefficient (PCC) of motif profiles between two symmetrical nucleosomal halves (positive and negative superhelical locations of nucleosomal DNA, please see Figure 2 of [10] for definition) for each TF and removed those with Pearson correlation coefficient values less than 0.4. Next, we applied t-distributed stochastic neighbor embedding (t-SNE) and projected all profiles onto two dimensions using the Rtsne function from the R package^48^. Then, the projected data were subjected to k-medoids clustering using the pam function from the R package with the optimal number of clusters equal to six (Supplementary Figure 6). The silhouette width is an estimate of the goodness of clusters, its values close to 1 correspond to a cluster where most objects are much closer to other objects in the same cluster than to other clusters. For each cluster, members with silhouette width <= 0.25 were considered as outliers and removed.

Since Micrococcal Nuclease has a sequence bias and cleaves DNA upstream of A or T more often than for G or C nucleotides, a certain nucleotide preference exists around the ends of nucleosomal DNA reads produced by the MNase-seq experiments. It may potentially bias the TF binding profiles near the nucleosomal DNA ends. Therefore, in our analysis, we excluded regions near the nucleosomal DNA ends. To identify binding modes between TFs and nucleosomes, we clustered binding motif profiles of different TFs centered at the nucleosomal dyad locations (+/− 60 base pair from dyad) using k-medoids clustering and those profiles fell into six distinct clusters (Figure 4).

## Results

### Transcription factor binding motif enrichment analysis can identify pioneer factors

Nucleosomes are generally considered as an impediment to the binding of TFs to DNA and thus binding sites of TFs are typically depleted in DNA regions with high nucleosome occupancy. However, pioneer TFs can recognize their binding motifs on nucleosomal DNA and trigger the opening of chromatin to recruit other TFs in a cell-type-specific fashion^2,49^.

Therefore, we hypothesized that DNA binding sites of pioneer factors should not be depleted and, in some cases should be enriched on nucleosome regions (NRs). At the same time, those canonical transcription factors that can bind only to the free DNA should exhibit the depletion of binding sites on nucleosome footprint regions and enrichment in nucleosome-free regions. To quantitively assess these trends, we have performed binding motif enrichment analysis for each of the 225 transcription factors and calculated the motif enrichment score (Supplementary Table 5 and 6). We have further analyzed the enrichment of TF binding sites on NRs in the differential open chromatin regions (see Methods for definition) and prioritized their ability to open the closed chromatin regions.

We selected 16 known pioneer factors from the literature (which could also be found in our list of 225 TFs) as positives (Supplementary Table 7) and other transcription factors were considered as negatives for this test. As we show in the next section, the negative set may also contain pioneer factors, therefore classification accuracy values provided below can be considered as a lower bound estimate. The enrichment score of the known PTFs was found to be significantly higher than for other factors (p-value = 0.0068 for all TFs and 0.002 for highly expressed TFs, Mann Whitney U test, Supplementary Figure 3).

**Figure 2.**
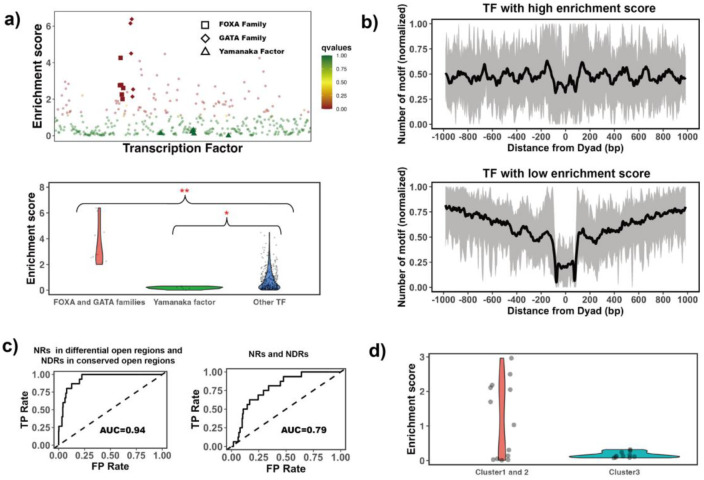
Identifying pioneer factors using motif enrichment analysis. a) Ranking of transcription factors by binding motif enrichment scores. Known pioneer factors from FOXA, GATA and Yamanaka factor families are indicated by squares, diamonds and triangles, while other TFs are shown as circles. Colors corresponds to false discovery rate (FDR) q-values. Mann–Whitney U tests are performed for FOXA, GATA factors and Yamanaka factors under the null hypothesis that their mean values of enrichment scores are equal to canonical TFs. * - p-value < 0.05; ** - p-value < 0.005. b) Binding motif profile of TFs with the highest and lowest motif enrichment scores (ranked at the top 20% among all TFs). The number of motifs for each TF is normalized within the range between 0 and 1 as follows: X(i)_normalized_) = (X(i) −X_min_)/(X_max_ −X_min_), X(i) is the number of binding motifs at the *i*_th_ base pair from the nucleosomal dyad position; X_max_ and X_min_ represent the maximal and minimal motif counts. c) TF motif enrichment score is used to distinguish PTFs (FOXA, GATA, and CEBP families) from other canonical TFs. Receiver operating curves (ROC) analyses of motif enrichment scores are performed. Here NRs (nucleosomal regions) are defined as nucleosomal DNA regions located in differentially open and NDRs (nucleosome-depleted regions) are located in conserved open chromatin regions. Using differential and conserved open chromatin regions in motif enrichment analysis significantly increased the AUC from 0.79 to 0.94. d) Comparison of the enrichment score of TFs in different clusters identified from recent EMSA experiments. Cluster 1: strong binders to both free DNA and nucleosomal DNA; Cluster 2: weak binders to both free DNA and nucleosomal DNA; Cluster 3: strong binders to free DNA but weak binders to nucleosomal DNA.

We have further used several approaches to validate our predictions. The first validation pertained to the ability of pioneer factors to open closed chromatin and therefore we hypothesize that the enrichment score calculated based on NRs located in differential open chromatin regions and NDRs located in conserved open chromatin regions will perform better in classification than the enrichment score using NRs and NDRs in either closed or open chromatin. As can be seen from Figure 2c, it is indeed the case, and the classification accuracy increases from AUC=0.79 to 0.94 upon the inclusion of differentially open regions (Mathew’s correlation coefficient increased from 0.2 to 0.3). Therefore, we used differential and conserved open chromatin regions in our further motif enrichment analyses. We should mention that by using differential and conserved open regions between H1 embryonic cell lines and other four somatic cell lines, we might skew our predictions to those PTFs which are more specific to H1 embryonic cell differentiation.

The second validation pertained to the known function of pioneer factors in cellular reprogramming and development. We found that the known pioneer factors that acted as key regulators of embryonic stem cell differentiation had the highest enrichment scores in our ranking (Figure 2a). These cases included pioneer factors from FOXA, GATA, and CEBP families^50–52^. In contrast, Yamanaka pioneer factors such as POU5F1 (OCT4), KLF4 and c-MYC, known for their roles in reprogramming somatic cells into induced pluripotent stem cells^53^, were strongly depleted at nucleosomes (Figure 2a). It has been previously shown that Yamanaka pioneer factors might target partial sequence motifs on nucleosomal DNA and require other factors for their binding to nucleosomes^16^ and therefore their enrichment score might not be expected to be high. On the other hand, we also found the known pioneer factors with relatively low enrichment scores including NFYA, NFYB and ESRRB (Supplementary Figure 4). These transcription factors regulate stem cell proliferation and maintenance of stem cell identity^54–56^ (Supplementary Table 7). Probably, the enrichment score calculated based on differentially open chromatin regions cannot distinguish them from other TFs.

Third, we performed another validation using recent data from high-throughput protein microarray and electromobility shift assays (EMSA) experiments on human TFs which systematically assessed transcription factor binding preferences to nucleosomal DNA versus free DNA^18^. The authors classified all studied transcription factors with respect to their strength of nucleosome binding into three clusters: strong binders to both free DNA and nucleosomal DNA (cluster 1); weak binders to both free DNA and nucleosomal DNA (cluster 2); strong binders to free DNA but weak binders to nucleosomal DNA (Cluster 3). TFs in cluster 3 have the lowest enrichment scores although this trend is not statistically significant (p-value = 0.22). As to TFs in cluster 1, half of them have binding sites enriched on nucleosome regions (known pioneer factors FOXA1, GATA4 and CEBPA) which correspond to strong nucleosome binders, and another half have binding sites enriched on nucleosome depleted regions. (Figure 2d)

### Prediction of transcription factors as potential nucleosome binders

Using gene expression data from the Roadmap Epigenomics Program, we identified 40 TFs in K562, HepG2 and HeLa cell lines that had their DNA binding sites significantly enriched on nucleosomal DNA of differentially open chromatin regions and were highly expressed in corresponding cell lines (Supplementary Table 6). Such transcription factors may preferentially target nucleosomes in closed chromatin regions and their binding may establish accessible chromatin. Among these 40 TFs, eight were well-characterized PTFs including GATA, FOXA and CEBP factors (Supplementary Table 7). To validate the remaining 32 PTF predictions, we performed literature search and found 14 transcription factors annotated in the literature as potential pioneer factors and/or potential nucleosome binders (Supplementary Table 8). For instance: HNF4A was annotated as a potential pioneer factor active in chromatin remodeling in the liver^57^; LEF1 was identified as a regulatory high mobility group (HMG) protein that could bind with nucleosome core particles^58^; CUX1 could specifically interact with its recognition motif in a nucleosomal context^59^; and JUN (JUNB and JUND), FOS (FOS and FOSL2), ATF (ATF4) and MAF (MAFG and MAFF) factors were identified as components of the AP-1 dimeric transcription complexes and were suggested to possess pioneer activity and to bind to nucleosome-occluded enhancers^60^ and trigger chromatin structure changes^61,62^.

### Association between binding motif profiles and nucleosome occupancy for pioneer factors

**Figure 3.**
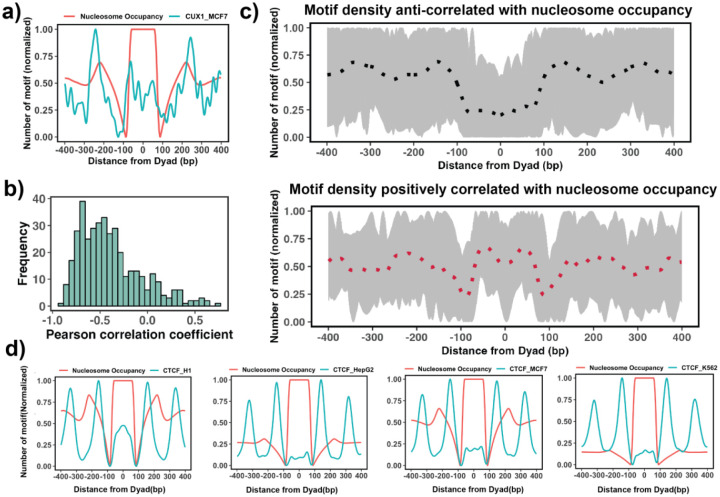
Association between the number of TF binding motifs (motif density) and nucleosome occupancy. a) A number of TF binding motifs and nucleosome occupancy values as a function of distance in base pairs from the nucleosome dyad. CUX1 in MCF-7 cell line is shown as an example. Nucleosome occupancy is calculated as the number of mapped nucleosomal DNA base pairs at each location using the MNase-seq data. Then the number of binding motifs at each location was normalized within the range between 0 and 1. b) Pearson correlation coefficients between the number of TF binding motifs and nucleosome occupancy values for each TF (n=225) with the median value of −0.46. c) Binding motif profiles of TFs with positive (red) or negative correlation coefficients (black) between the number of binding motifs and nucleosome occupancy. Dashed lines represent the average of the binding motif profiles. d) Comparison of binding motif profiles of CTCF between H1 embryonic stem cell line and other somatic cell lines.

The enrichment analysis described above is the first step to estimate the tendency of transcription factor binding sites to be located on nucleosomal footprints. The next step is to take into account the actual locations of binding sites on the nucleosomal footprint with respect to the nucleosome dyad. To this end, we tested if there is a significant association between binding motif density (see Methods) and nucleosome occupancy values (Figure 3a). Our results show that 87% TFs have negative Pearson correlation coefficients between binding motif density and nucleosome occupancy values (Figure 3b) which is consistent with the fact that nucleosomes generally restrict the access of TFs to their binding sites on DNA molecules.

We have also identified 37 transcription factors that have a statistically significant positive correlation between binding motif densities and nucleosome occupancy and may also be classified as potential nucleosome binders (Supplementary Table 9 and Figure 3). These cases include known PTFs such as POU5F1(OCT4), GATA3, CEBPB, NFYA and NFYB (Supplementary Table 9) while 11 factors with high positive correlation coefficients (PCC >= 0.3) that were not in our true positive list of 16 known PTFs (see previous section). As can be seen in Supplementary Table 9, with the exception of a few cases, none of these transcription factors has been identified as being pioneer factors in all cell types because pioneer activity is often cell-type specific. For example, CTCF was identified as having a significant correlation between binding motifs and nucleosome occupancy for the human embryonic stem cell line (PCC = 0.1, p-values < 0.05), but not for other somatic cell lines (Figure 3d). Indeed, previous studies have indicated that CTCF proteins could access the binding sites in nucleosomes and may function as a pioneer factor in embryonic stem cells but to a lesser extent in differentiated cells^63–65^.

### Deciphering the interaction modes between transcription factors and nucleosomes

Using NCAP–SELEX approach, a recent study characterized the interaction landscape between pioneer transcription factors and nucleosomes and revealed different binding modes of pioneer factors: DNA end binding, dyad binding, gyre binding and periodic binding^14^. To compare TF motif profiles with NCAP–SELEX data, we calculated binding profiles of various TFs and filtered out low-quality profiles using the criteria described in the Methods section and then calculated motif enrichment scores for those binding modes identified by NCAP–SELEX approach. Due to the limited number of transcription factors observed in both our dataset and NCAP–SELEX study (24 transcription factors in total), we mainly focused on the DNA end binding (super helical locations (SHL) from +/− 5.5 to +/− 7) and dyad binding modes (SHL from 0 to +/− 1.5). To estimate the preferential binding of TFs to the ends of nucleosomal DNA compared to the nucleosomal dyad, we calculated the end/dyad binding ratio (*R*_*end/dyad*_) as the number of binding motifs at the DNA ends (SHLs from +/− 5.5 to +/− 7) divided by the number of binding motifs near dyad regions (SHLs from 0 to +/−1.5).

In NCAP-SELEX experimental analyses, to quantify the preference of PTF binding to nucleosomes^14^, the binding signals were compared by calculating the mutual information (MI) content between 3-mer distributions at two non-overlapping positions of the ligand, aimed at finding if SELEX ligand may contact these positions at the same time. Since nucleosomes can bind to most sequences, whereas TFs bind to only a few specific sequences, the NCAP-SELEX study calculated the enriched MI (E-MI) score to separate the TF signals from nucleosome signals by limiting the MI measure to the top 10 most enriched 3-mer pairs. E-MI penetration score corresponds to E-MI drop by half compared to the E-MI maximum and larger values pointed to the favorable binding to the dyad regions^14^. As our (*R*_*end/dyad*_) and experimental EMI penetration values are on opposite scale, we indeed observe a statistically significant negative correlation between (*R*_*end/dyad*_) and EMI penetration values (Pearson correlation coefficient = −0.45, p-value = 0.025). This shows that our computational analysis is able to capture the fine features of TF binding motifs on nucleosomes. For the EMI intensity, although a negative linear dependence trend was evident, the correlation was not significant (Supplementary Figure 5).

### Clustering reveals several groups of pioneer transcription factor binding sites

**Figure 4.**
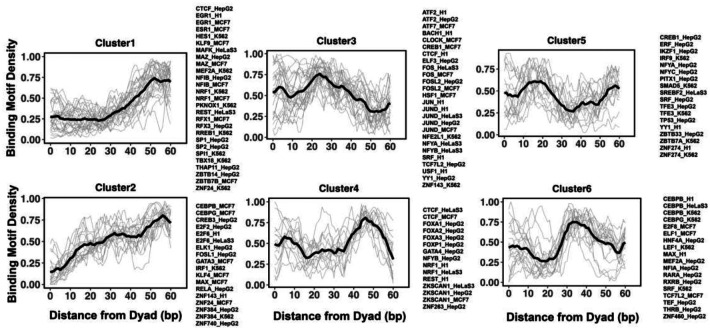
Clusters of TF binding motif profiles on nucleosomal DNA. Binding motif profiles centered at nucleosomal dyad locations (+/− 60 base pair from dyad) are clustered using k-medoids clustering with k=6. Binding motif profiles between two symmetrical nucleosomal halves are combined for each TF. The black line represents the averaged profiles of all TFs in the same cluster.

Previous studies indicated that while on average TF binding tends to act as a barrier for nucleosome positioning, there is a number of different signatures of mutual TF/nucleosome positioning^66^. Here we characterized such scenarios taking into account the pioneering properties of TFs. To pinpoint the details of PTF binding motifs at near-single nucleotide resolution on nucleosomal DNA, we have performed k-medoids clustering of TF binding motif profiles for all 225 transcription factors (Figure 4 and Supplementary Figure 6). Transcription factors in the first and second cluster show that motif density increases with the distance from the dyad. These clusters include cases of canonical factors or those PTF that preferentially interact with the ends of nucleosomal DNA (from super helical locations(SHL) +/− 5.5 to +/− 7, an illustration of SHLs on nucleosome structures is shown in Supplementary Figure 7), such as pioneer factors CEBPB and GATA3, consistent with the NCAP-SELEX data. In addition, it has been recently shown that GATA3 factor targets nucleosomal DNA around the SHL +/−5.5 position^22^. In cluster 4 and 6, TFs preferentially interact with the nucleosomal DNA around the dyad regions and SHL +/−4–5 and SHL +/−3–4, respectively, whereas transcription factors from cluster 3 and 5 preferentially occupy SHL +/−2–3 and SHL +/−1–2 respectively. All known pioneer factors from FOXA families belong to cluster 4 and it has been shown that FOXA1 can target its binding motifs on nucleosomal DNA, displace linker histones and mediate chromatin opening^67^. We can see many transcription factors from the same family or from different cell types assigned to the same cluster. There are a few exceptions, however (CREB1, CTCF, MEF2A, NFYA, NFYB and YY1) where the same transcription factor from different cell lines has been assigned to different clusters. Possible reasons include the cell-type-specific epigenetic marks recognized by pioneer transcription factors or cooperativity binding between factors differentially expressed in various cell types.

## Discussion

It is a subject of ongoing debate - how the modulation of chromatin accessibility with high spatiotemporal precision can be achieved in the nucleus. PTFs can infiltrate closed chromatin and directly recognize their binding sites on nucleosomes. There are about two thousand TFs in human and a few hundred PTFs among them. Yet, for the vast majority of human PTFs, the locations of their binding sites and mechanisms of binding and regulation remain unknown^18,68^. There are many different experimental assays providing information on transcription factor binding sites but they suffer from multiple drawbacks and often require a priori knowledge of the TFs being tested. PTFs are very dynamic, may target partial binding sites and work cooperatively with other factors which complicate their identification^49,69,70^. For example, the two recent Cryo-EM structures of pioneer factor SOX2-nucleosome complexes showed different mechanisms of binding^20,21^. Therefore, there is a pressing need to develop predictors and classifiers of human pioneer transcription factors.

The goal of this study has been to gain functional insights into the mechanisms of binding and infiltration of PTFs into chromatin at the level of nucleosomes. To reach this goal we used ChIP-seq, MNaseq-seq and DNase-seq data from the five different cell lines and 225 human transcription factors. We have developed a computational framework to systemically investigate the ability of transcription factors to bind to nucleosomes. We found that using the information on differentially open chromatin regions (open in one cell line, closed in another) and TF binding sites, the highest classification accuracy is achieved with AUC=0.94 in discriminating pioneers from canonical transcription factors. This finding supports the view that TF binding to nucleosomes leads to DNA and chromatin opening and correlates with the reprogramming potential^18^. Our study has verified the known and predicted new pioneer transcription factors as nucleosome binders in embryonic cell differentiations (Supplementary Table 6 and 7). These predicted cases include 32 highly expressed TFs without known pioneer activity and may be subjected to future experimental validations. As we integrated a large amount of data of relatively low resolution, we tried to verify it rigorously using strict criteria which possibly resulted in missing some pioneer factors. Our study has further identified six distinctive clusters of TF binding profiles with nucleosomal DNA. These clusters point to the diversity of binding motifs on nucleosomal DNA. Transcription factors belonging to the same cluster may exhibit potential competitive binding.

We should mention that our criteria of pioneer activity - significant enrichment on the nucleosomal DNA of differentially open chromatin regions and expression in the corresponding cell lines – has limitations. Indeed, interactions of PTFs with nucleosomes may depend on the binding of other factors, but deducing such dependencies from the current data is very cumbersome. In addition, PTF binding relies not only on specific recognition of DNA binding sites but can be governed by nucleosome dynamics and spontaneous DNA unwrapping. The latter processes are not uniform throughout the genome and depend on local nucleosomal DNA sequence, histone variant deposition and chromatin modifications. Such genomic-location specificity o f PTF activity is challenging to address. Finally, pioneer factors may exhibit multivalent binding recognizing not only DNA binding sites but also some parts of histone core or histone tails.

Nucleosomes represent hub points in epigenetic signaling pathways and identifying complex epigenetic relationships at the level of single nucleosomes may yield functional insights into the mechanisms of binding and infiltration of pioneer transcription factors into chromatin during differentiation and reprogramming. Transcription factors regulate a large number of signaling pathways and their dysregulation contributes to a plethora of human diseases, including diabetes, cardiovascular diseases and many cancers. Conventional transcription factors have been used for years as biomarkers and drug targets, however, the therapeutic potential of pioneer transcription factors is lagging behind. To fill this gap, integrative approaches using large-scale low- or medium-resolution data with precise molecular modeling or protein-protein docking may provide the required detailed characterization of many predicted PTFs in the future.

## Supplementary Material

Supplement 1
