# Supplementary material for: Detection of new pioneer transcription factors as cell-type specific nucleosome binders
Source: bioRxiv. 2023 May 12:2023.05.10.540098. Preprint. [Version 1] doi: 10.1101/2023.05.10.540098 (PMC10327179; doi:10.1101/2023.05.10.540098)
Supplement: Supplementary file 1 [file media-1.pdf]

Supplementary Table 1. Summary of MNase-seq experimental data for genome-wide nucleosome mapping.

Cell lines	Sequencing type	GEO Accession number
**MCF-7 breast cancer cell line**	paired-end	GSE51097
**K562 immortalized myelogenous leukemia cell line**	paired-end	GSE78984
**H1 human embryonic stem cell line**	paired-end	GSM1194220
**HepG2 human liver cancer cell line**	paired-end	GSM3718063
**HeLa cell line**	paired-end	GSE100401

Supplementary Table 2. Summary of the number of identified nucleosome regions (NRs) in each cell line.

Cell lines	number of NRs
**MCF-7 breast cancer cell line**	10290828
**K562 immortalized myelogenous leukemia cell line**	2780423
**H1 human embryonic stem cell line**	12551429
**HepG2 human liver cancer cell line**	5193194
**HeLa cell line**	9842040

Supplementary Table 3. List of analyzed TFs and the correspond IDs of position frequency matrices (PFMs) files in the JASPER database.

**TF gene name**	**Motif ID**	**TF gene name**	**Motif ID**	**TF gene name**	**Motif ID**	**TF gene name**	**Motif ID**
**ATF2**	MA1632.1	**GABPA**	MA0062.3	**MYBL2**	MA0777.1	**SPDEF**	MA0686.1
**ATF3**	MA0605.2	**GATA1**	MA0035.4	**MYC**	MA0147.3	**SPI1**	MA0080.5
**ATF4**	MA0833.2	**GATA2**	MA0036.3	**NEUROD1**	MA1109.1	**SREBF1**	MA0595.1
**ATF6**	MA1466.1	**GATA3**	MA0037.3	**NFATC3**	MA0625.1	**SREBF2**	MA0596.1
**ATF7**	MA0834.1	**GATA4**	MA0482.2	**NFE2**	MA0841.1	**SRF**	MA0083.3
**BACH1**	MA1633.1	**GFI1**	MA0038.2	**NFE2L1**	MA0089.2	**STAT3**	MA0144.2
**BCL6**	MA0463.2	**GMEB2**	MA0862.1	**NFE2L2**	MA0150.1	**TBX18**	MA1565.1
**BHLHE40**	MA0464.2	**HES1**	MA1099.2	**NFIA**	MA0670.1	**TBX2**	MA0688.1
**CEBPA**	MA0102.4	**HEY1**	MA0823.1	**NFIB**	MA1643.1	**TBX3**	MA1566.1
**CEBPB**	MA0466.2	**HINFP**	MA0131.2	**NFIC**	MA0161.2	**TCF3**	MA0522.3
**CEBPD**	MA0836.2	**HLF**	MA0043.3	**NFIL3**	MA0025.2	**TCF7**	MA0769.2
**CEBPG**	MA0838.1	**HMBOX1**	MA0895.1	**NFIX**	MA0671.1	**TCF7L2**	MA0523.1
**CLOCK**	MA0819.1	**HNF1A**	MA0046.2	**NFKB2**	MA0778.1	**TCFL5**	MA0632.2
**CREB1**	MA0018.4	**HNF1B**	MA0153.2	**NFYA**	MA0060.3	**TEAD1**	MA0090.3
**CREB3**	MA0638.1	**HNF4A**	MA0114.4	**NFYB**	MA0502.2	**TEAD2**	MA1121.1
**CREB3L1**	MA0839.1	**HNF4G**	MA0484.2	**NFYC**	MA1644.1	**TEAD4**	MA0809.2
**CREM**	MA0609.2	**HOXA5**	MA0158.2	**NKX3-1**	MA0124.1	**TEF**	MA0843.1
**CTCF**	MA0139.1	**HSF1**	MA0486.2	**NR2C1**	MA1535.1	**TFAP4**	MA0691.1
**CTCFL**	MA1102.2	**HSF2**	MA0770.1	**NR2C2**	MA0504.1	**TFCP2**	MA0145.3
**CUX1**	MA0754.1	**IKZF1**	MA1508.1	**NR2F1**	MA0017.2	**TFDP1**	MA1122.1
**DBP**	MA0639.1	**IRF1**	MA0050.2	**NR2F2**	MA1111.1	**TFE3**	MA0831.2
**DLX6**	MA0882.1	**IRF2**	MA0051.1	**NR3C1**	MA0113.3	**TF_name**	Motif_ID
**E2F1**	MA0024.3	**IRF3**	MA1418.1	**NR4A1**	MA1112.2	**TGIF2**	MA0797.1
**E2F2**	MA0864.2	**IRF5**	MA1420.1	**NR5A1**	MA1540.1	**THAP1**	MA0597.1
**E2F3**	MA0469.3	**IRF9**	MA0653.1	**NRF1**	MA0506.1	**THAP11**	MA1573.1
**E2F4**	MA0470.2	**ISL2**	MA0914.1	**NRL**	MA0842.2	**THRB**	MA1574.1
**E2F6**	MA0471.2	**ISX**	MA0654.1	**ONECUT1**	MA0679.2	**TP53**	MA0106.3
**E2F7**	MA0758.1	**JUN**	MA0488.1	**ONECUT2**	MA0756.1	**USF1**	MA0093.3
**E2F8**	MA0865.1	**JUNB**	MA0490.2	**OVOL1**	MA1544.1	**USF2**	MA0526.3
**EGR1**	MA0162.4	**JUND**	MA0491.2	**PBX2**	MA1113.2	**VEZF1**	MA1578.1
**ELF1**	MA0473.3	**KLF10**	MA1511.1	**PITX1**	MA0682.2	**XBP1**	MA0844.1
**ELF2**	MA1483.1	**KLF11**	MA1512.1	**PKNOX1**	MA0782.2	**YY1**	MA0095.2
**ELF3**	MA0640.2	**KLF13**	MA0657.1	**POU5F1**	MA1115.1	**ZBED1**	MA0749.1
**ELF4**	MA0641.1	**KLF16**	MA0741.1	**PPARG**	MA0066.1	**ZBTB12**	MA1649.1

**ELK1**	MA0028.2	**KLF4**	MA0039.4	**PRDM1**	MA0508.3	**ZBTB14**	MA1650.1
**ELK3**	MA0759.1	**KLF6**	MA1517.1	**PRDM4**	MA1647.1	**ZBTB26**	MA1579.1
**ELK4**	MA0076.2	**KLF9**	MA1107.2	**RARA**	MA0729.1	**ZBTB33**	MA0527.1
**ERF**	MA0760.1	**LBX2**	MA0699.1	**RBPJ**	MA1116.1	**ZBTB7A**	MA0750.2
**ESR1**	MA0112.3	**LEF1**	MA0768.1	**RELA**	MA0107.1	**ZBTB7B**	MA0694.1
**ESRRA**	MA0592.3	**MAFF**	MA0495.3	**REST**	MA0138.2	**ZEB1**	MA0103.3
**ESRRB**	MA0141.3	**MAFG**	MA0659.2	**RFX1**	MA0509.2	**ZKSCAN1**	MA1585.1
**ETS1**	MA0098.3	**MAFK**	MA0496.3	**RFX3**	MA0798.2	**ZKSCAN5**	MA1652.1
**ETS2**	MA1484.1	**MAX**	MA0058.3	**RFX5**	MA0510.2	**ZNF143**	MA0088.2
**ETV1**	MA0761.2	**MAZ**	MA1522.1	**RFX7**	MA1554.1	**ZNF148**	MA1653.1
**ETV4**	MA0764.2	**MEF2A**	MA0052.4	**RORA**	MA0071.1	**ZNF24**	MA1124.1
**ETV5**	MA0765.2	**MEF2D**	MA0773.1	**RREB1**	MA0073.1	**ZNF263**	MA0528.2
**ETV6**	MA0645.1	**MEIS1**	MA0498.2	**RUNX1**	MA0002.1	**ZNF274**	MA1592.1
**FOS**	MA0476.1	**MEIS2**	MA0774.1	**RXRB**	MA0855.1	**ZNF282**	MA1154.1
**FOSL1**	MA0477.2	**MGA**	MA0801.1	**SIX1**	MA1118.1	**ZNF317**	MA1593.1
**FOSL2**	MA0478.1	**MITF**	MA0620.3	**SMAD3**	MA0795.1	**ZNF382**	MA1594.1
**FOXA1**	MA0148.4	**MIXL1**	MA0662.1	**SMAD5**	MA1557.1	**ZNF384**	MA1125.1
**FOXA2**	MA0047.3	**MLX**	MA0663.1	**SNAI1**	MA1558.1	**ZNF460**	MA1596.1
**FOXA3**	MA1683.1	**MNT**	MA0825.1	**SOX13**	MA1120.1	**ZNF652**	MA1657.1
**FOXK1**	MA0852.2	**MNX1**	MA0707.1	**SOX18**	MA1563.1	**ZNF740**	MA0753.2
**FOXK2**	MA1103.2	**MSX2**	MA0708.1	**SP1**	MA0079.4	**ZSCAN29**	MA1602.1
**FOXO4**	MA0848.1	**MTF1**	MA0863.1	**SP2**	MA0516.2		
**FOXP1**	MA0481.3	**MXI1**	MA1108.2	**SP4**	MA0685.1		

Supplementary Table 4. Contingency table for binding enrichment analysis to quantify TF's binding preferences on NRs or NDRs. Nucleosome regions (NRs) were determined as genomic regions centered at representative dyad positions and flanked by 73 bp segments on each side. Nucleosome-depleted regions (NDRs) represented the genomic regions free of nucleosomes and were identified as genomic regions not covered by any mono-nucleosome fragments (120 to 180 base pairs length) in MNase-seq data. For motif enrichment analysis of transcription factors, we selected the NDRs located in the open chromatin regions. We used two sets of NRs and NDRs for enrichment analysis, one is the NRs located in differential open chromatin regions and NDRs located in conserved open chromatin regions while another is all NFRs and NDRs. The first set performed better in the classification of TFs.

	**# of base pairs overlapped with ChIP-seq binding motifs**	**# of base pairs outside of ChIP-seq binding motifs**
**Nucleosome Regions**	a	b
**Nucleosome Depleted Regions**	c	d

Supplementary Table 5. TFs that have binding sites enriched on NRs located in the differential open chromatin regions (Enrichment score >=1 and q-value <=0.05).

TF name	Cell line	Enrichment score	TF name	Cell line	Enrichment Score
**ATF4**	K562	1.44	**MAFF**	HeLa-S3,HepG2,K562	1.38,1.25,2.35
**BACH1**	K562	2.20	**MAFG**	HepG2,K562	1.28,2.79
**BCL6**	HepG2	1.17	**MAFK**	K562	1.90
**CEBPA**	HepG2	2.95	**MEF2A**	HepG2,K562	1.95,2.51
**CEBPB**	HeLa-S3,HepG2,K562	2.58,1.68,1.26	**MEF2D**	K562	3.11
**CEBPD**	HepG2	2.84	**MEIS2**	K562	1.11
**CEBPG**	HepG2,K562,MCF-7	1.92,1.29,1.35	**NFATC3**	K562	1.76
**CUX1**	K562	1.29	**NFE2**	K562	2.28
**ESRRA**	HepG2	1.42	**NFE2L1**	K562	2.75
**FOS**	HeLa-S3,K562,MCF-7	1.25,1.91,1.78	**NFIC**	HepG2	1.86
**FOSL1**	K562	1.89	**NFIL3**	HepG2	1.80
**FOSL2**	HepG2,MCF-7	1.46,1.76	**NFIX**	K562	1.46
**FOXA1**	HepG2,K562,MCF-7	2.76,4.27,2.78	**NR2F1**	HepG2,K562	1.61,1.08
**FOXA2**	HepG2	2.26	**NR2F2**	HepG2,K562	2.70,1.75
**FOXA3**	HepG2,K562	2.01,2.61	**NR4A1**	K562	1.87
**FOXK2**	K562	1.35	**NR5A1**	HepG2	1.81
**FOXP1**	HepG2	2.38	**ONECUT1**	HepG2	1.72
**GATA1**	K562	6.15	**ONECUT2**	HepG2	2.33
**GATA2**	HepG2,K562	4.51,6.39	**PBX2**	HepG2,K562	1.70,1.87
**GATA3**	MCF-7	2.14	**PPARG**	HepG2	1.13
**GATA4**	HepG2	2.54	**PRDM1**	HeLa-S3	1.60
**GFI1**	HepG2	1.14	**RARA**	HepG2	1.95
**HLF**	HepG2	1.41	**RUNX1**	K562	2.93
**HMBOX1**	K562	1.55	**RXRB**	HepG2	2.03
**HNF1A**	HepG2	4.09	**SOX13**	HepG2	2.43
**HNF1B**	HepG2	1.55	**SRF**	K562	2.05
**HNF4A**	HepG2	3.64	**STAT3**	HeLa-S3	4.25
**HNF4G**	HepG2	3.32	**TCF7**	HepG2,K562	3.51,1.30
**IKZF1**	K562	1.35	**TCF7L2**	HepG2,MCF-7	2.73,1.42
**IRF1**	K562	1.31	**TEAD1**	HepG2	1.36
**IRF9**	K562	2.57	**TEF**	HepG2	1.61
**JUN**	HeLa-S3	1.22	**THRB**	HepG2	2.15
**JUNB**	K562	2.06	**ZNF24**	K562	1.13
**JUND**	HeLa-S3,HepG2,K562	2.27,1.44,1.97	**ZNF652**	HepG2	1.30
**LEF1**	K562	4.48			

Supplementary Table 6. TFs that have binding sites enriched on NRs in the differential open chromatin regions (Enrichment score >=1 and q-value <=0.05) and are highly expressed (RPKM >=10) in corresponding cell lines.

TF name	Cell line	Enrichment score	Expression level (RPKM value)
**ATF4**	K562	1.44	374.61
**BACH1**	K562	2.20	21.38
**BCL6**	HepG2	1.17	28.63
**CEBPA**	HepG2	2.95	33.37
**CEBPB**	HeLa-S3,HepG2,K562	2.58,1,68,1.26	29.70,42.83,10.53
**CEBPD**	HepG2	2.84	26.11
**CEBPG**	HepG2,K562	1.92,1.29	31.21,33.14
**ESRRA**	HepG2	1.42	16.10
**FOS**	HeLa-S3	1.25	11.02
**FOSL2**	HepG2	1.46	19.26
**FOXA1**	HepG2	2.76	32.38
**FOXA2**	HepG2	2.26	10.88
**FOXA3**	HepG2,K562	2.01,2.61	57.20
**FOXK2**	K562	1.35	22.64
**GATA1**	K562	6.15	125.16
**GATA2**	K562	6.39	18.36
**GATA4**	HepG2	2.54	13.42
**HLF**	HepG2	1.41	180.96
**HMBOX1**	K562	1.55	27.41
**HNF4A**	HepG2	3.64	72.69
**IKZF1**	K562	1.35	14.53
**JUNB**	K562	2.06	19.14
**JUND**	HeLa-S3,HepG2,K562	2.27,1.44,1.97	14.06,46.02,41.06
**LEF1**	K562	4.48	13.49
**MAFF**	HepG2	1.25	13.95
**MAFG**	HepG2,K562	1.28,2.79	21.66,26.12
**MEF2A**	HepG2	1.95	14.56
**NFATC3**	K562	1.76	56.33
**NFE2**	K562	2.28	143.18
**NFE2L1**	K562	2.75	28.47
**NFIL3**	HepG2	1.80	51.02
**NR2F1**	HepG2	1.61	28.15
**NR5A1**	HepG2	1.81	28.13
**PBX2**	HepG2,K562	1.70,1.87	37.77,49.68

**PPARG**	HepG2	1.13	26.05
**RXRB**	HepG2	2.03	34.15
**SRF**	K562	2.05	28.71
**STAT3**	HeLa-S3	4.25	25.54
**TEAD1**	HepG2	1.36	13.31
**ZNF24**	K562	1.13	53.34

Supplementary Table 7. Known pioneer transcription factors (PTFs) and their function.

Transcription Factor Name	Function
FOXA1, FOXA2, FOXA3	Tissue-specific gene activation; embryonic development, establishment of tissue-specific gene expression and regulation of gene expression in differentiated tissues.
GATA1, GATA2, GATA3, GATA4	Early stages of cell differentiation and organ development across a variety of tissues; development and maintenance of hematopoietic systems.
CEPBA, CEBPB	Fate decisions during myeloid differentiation; essential for maintaining homeostasis of both embryonic and adult tissues; key regulators of hepatocyte differentiation.
NFYA, NFYB	Cell type-specific master transcription factors; maintenance of embryonic stem cell (ESC) identity.
ESRRB	Suppress cell differentiation and sustain ESC self-renewal
POU5F1 (OCT-4)	Yamanaka transcription factors; cellular reprogramming of somatic cells into induced pluripotent stem cells.
KLF4	
NEUROD1	Neuronal reprogramming/neuronal conversion from human fibroblasts; neuronal differentiation.
TP53	DNA repair, cell-cycle arrest, and apoptosis; tumor suppressor.

Supplementary Table 8. List of TFs that have been previously suggested as potential pioneer factors or nucleosome binders from literature.

TF name	Function	Reference
LEF1	able to bind with HIV-1 nucleosome core particles	1
HNF4A	a potential pioneer factor in remodeling the active chromatin landscape in the liver	2
JUNB	a component of pioneer factor AP-1 dimeric transcription complexes	3,4
JUND	a component of pioneer factor AP-1 dimeric transcription complexes	3,4
PBX2	one PBC transcription factor which could have pioneer activity	5
NR5A1	considered as a pioneer transcription factor for Leydig cell development and function	6
NFE2	interact with the cognate motif on the nucleosome before chromatin is remodeled	7
ATF4	potentially act as a pioneer factor to alter chromatin structure	8
CUX1	specifically interacts with its recognition motif in a nucleosomal context without displacing the nucleosome core	9
FOXK2	possibly acts as an early pioneer factor in gene activation during cell differentiation	10
FOS	a component of pioneer factor AP-1 dimeric transcription complexes	3,4
FOSL2	a component of pioneer factor AP-1 dimeric transcription complexes	3,4
MAFG	a component of pioneer factor AP-1 dimeric transcription complexes	3,4
MAFF	a component of pioneer factor AP-1 dimeric transcription complexes	3,4

Supplementary Table 9. List of TFs with positive correlations between the number of binding sites and nucleosome occupancy levels (PCC >=0 and p-value <=0.05).

TF name	Cell Line	PCC
**ZKSCAN1**	HeLaS3, K562, MCF7, HepG2	0.75,0.64,0.63, 0.34
**ESR1**	MCF7	0.58
**NFATC3**	K562	0.56
**ZBTB7B**	MCF7, HepG2	0.55,0.37
**MAX**	H1	0.51
**TBX2**	HepG2	0.50
**NFIB**	MCF7	0.48
**USF1**	H1	0.46
**CUX1**	MCF7, K562	0.41,0.08
**ZNF274**	HepG2	0.37
**SREBF1**	MCF7	0.36
**NFYB**	HeLaS3	0.33
**ZNF24**	MCF7, K562	0.32,0.08
**NR2F2**	MCF7	0.31
**ZNF282**	K562	0.30
**RELA**	K562	0.30
**USF2**	HeLaS3	0.28
**ESRRA**	MCF7	0.28
**POU5F1**	H1	0.26
**NFKB2**	HepG2	0.22
**BACH1**	H1	0.22
**SREBF2**	HeLaS3	0.21
**TEAD4**	H1	0.20
**MIXL1**	HepG2	0.18
**NR4A1**	K562	0.15
**E2F6**	H1	0.15
**RARA**	HepG2	0.14
**CEBPB**	HeLaS3	0.13
**SMAD5**	K562	0.12
**NFYA**	HeLaS3	0.11
**HMBOX1**	K562	0.11
**ZNF460**	HepG2	0.11
**CTCF**	H1	0.10
**EGR1**	H1	0.10
**GATA3**	MCF7	0.09

**ATF2**	K562	0.08
**TCF7L2**	MCF7	0.07

Supplementary Figure 1. Identification of dinucleotide patterns of nucleosomal DNA (MCF7 cell line is shown as one representative case).

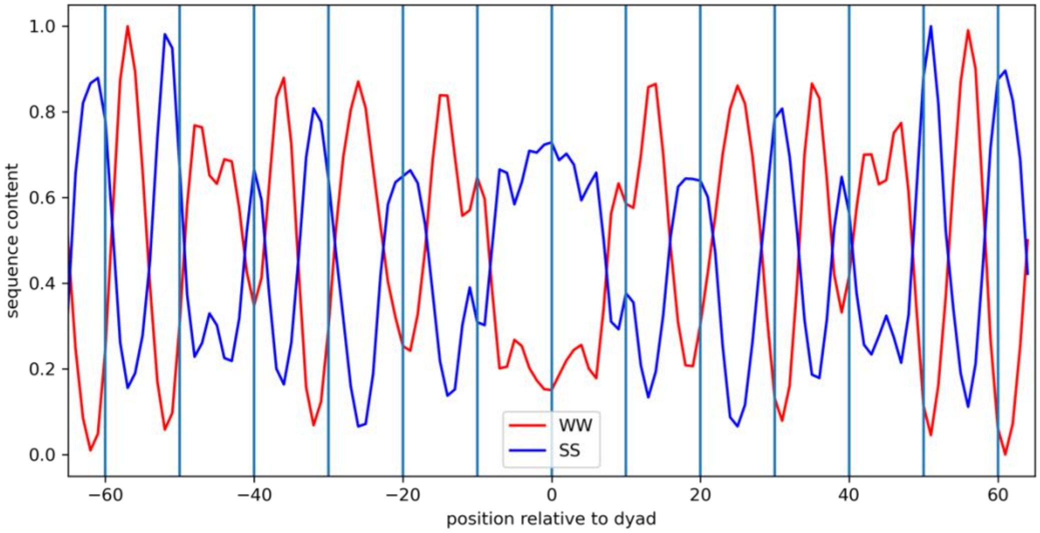

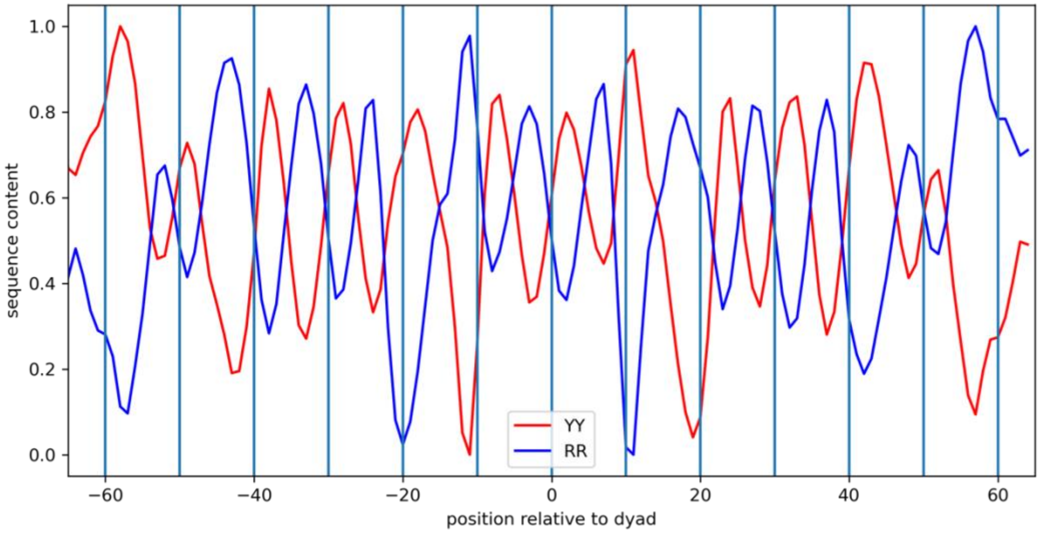

Supplementary Figure 2. Calculation of binding motif profile for TF. Arrows indicate the mapped ChIP-seq motifs to the closest dyad. For each asymmetric(Non-palindromic) TF motif, we considered both the motif sequence and its sequence on a complementary strand. Then, the number of motifs at each location on nucleosomal DNA and flanking DNA regions was counted to derive the binding motif profile.

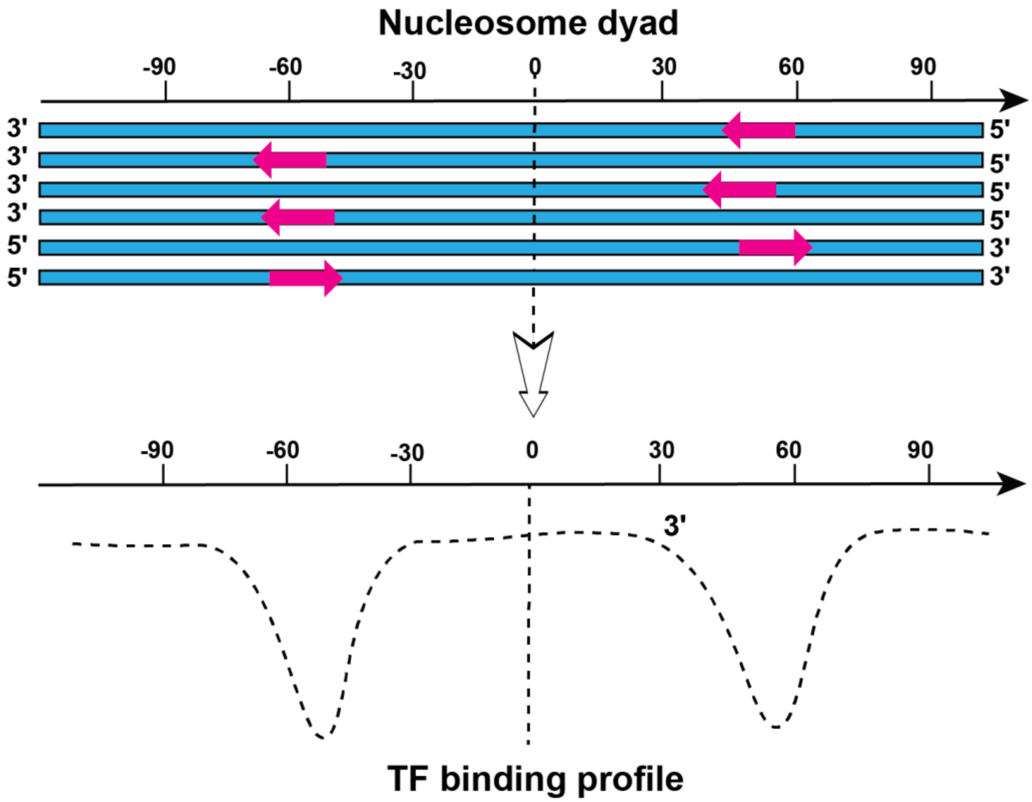

Supplementary Figure 3. Ranking of TFs from motif enrichment analysis calculated with NRs located in differential open chromatin regions and NDRs located in conserved open chromatin regions. PTFs and non-PTFs are shown with red and light blue colors. Mann–Whitney U test is performed under the null hypothesis that the mean values of enrichment scores of two groups are equal. a) and b) show the ranking of all TFs and significantly expressed TFs by their enrichment score.

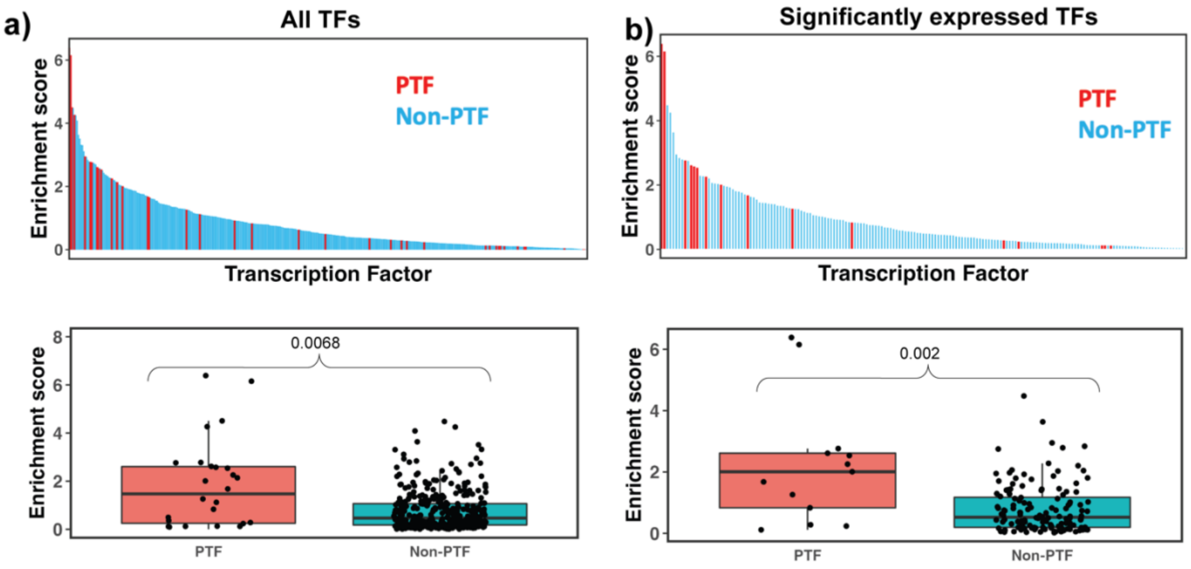

Supplementary Figure 4. Comparison of the enrichment scores of pioneer factors NFYA, NFYB and ESRRB (highlighted as red and green) with other TFs. Mann–Whitney U test is performed under the null hypothesis that the mean values of enrichment scores of two groups are equal.

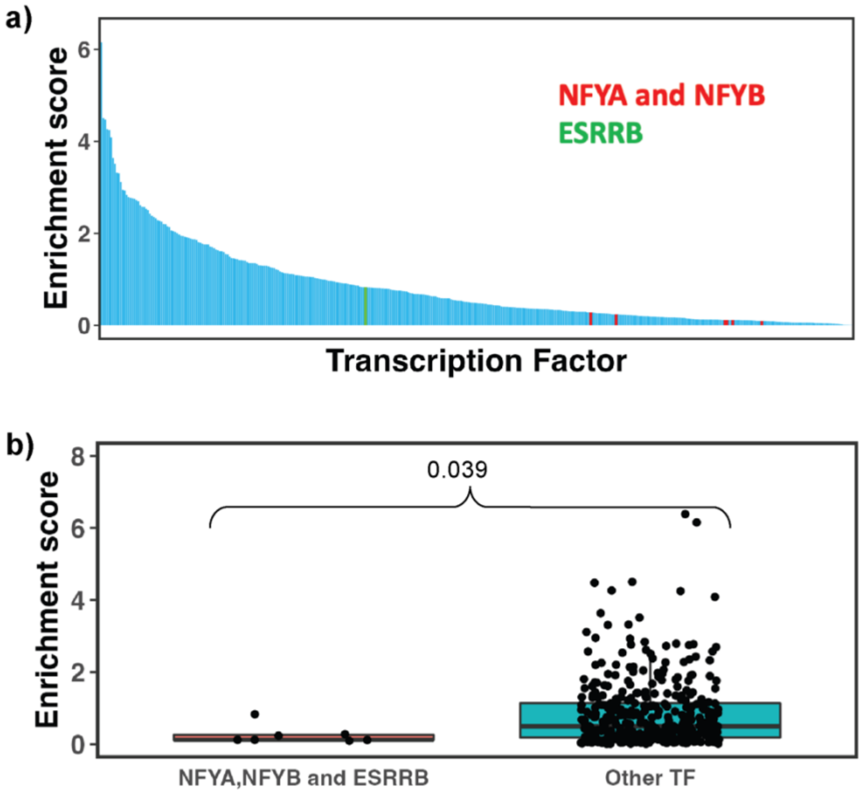

Supplementary Figure 5. Correlation between calculated TF end/dyad binding ratio ($\tau_{end/dyad}$) from our study and the EMI intensity and EMI penetration values from recent nucleosome NCAP–SELEX experiments[11].

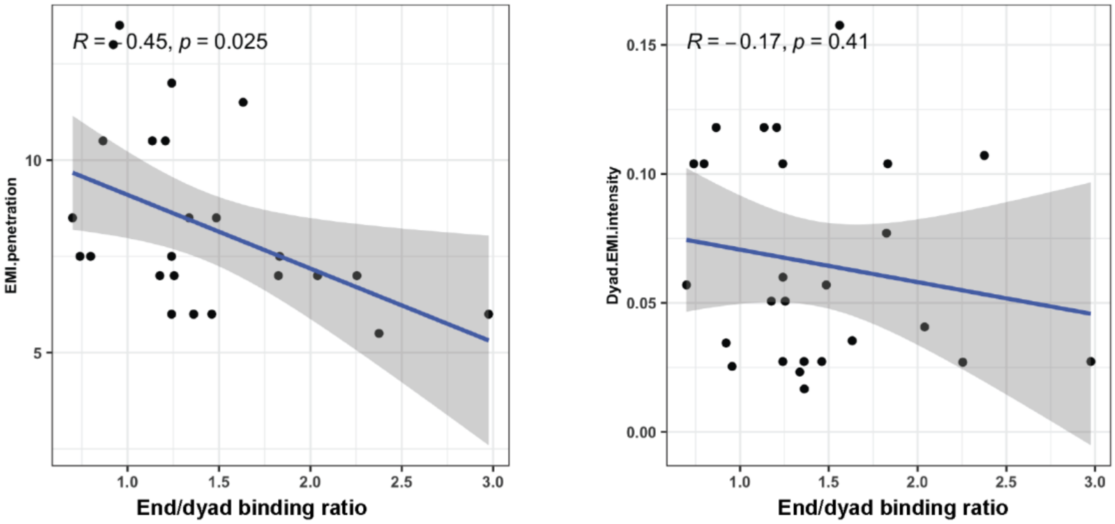

Supplementary Figure 6. a) Variation of average silhouette width with the number of clusters in k-medoids clustering of TF binding profiles. Six is selected as an optimal number of clusters. b) Distribution of silhouette width for all points (S_i) in each cluster. Each point with S_i <=0.25 is considered as outlier and excluded from the analysis. c) Pearson correlation coefficients (PCCs) of binding motif profiles between two symmetrical nucleosomal halves (define SHL) of all TFs. TFs with PCC values less than 0.4 and the total number of base pairs of binding motifs in NRs less than 500 are excluded from the analysis.

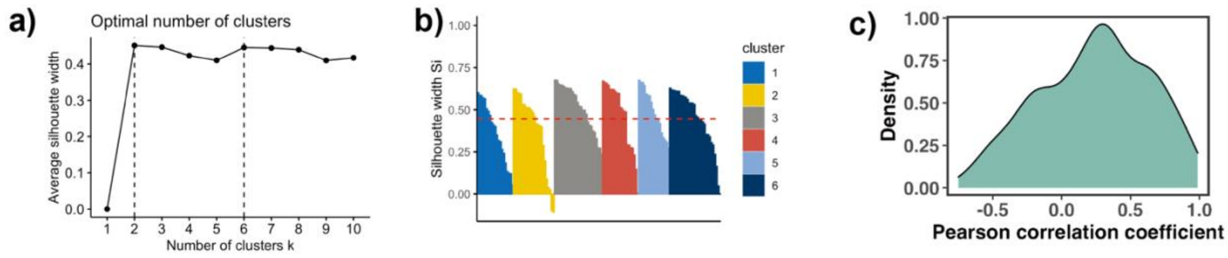

Supplementary Figure 7. Illustrations of superhelical locations (SHLs) on nucleosome structures. The DNA locations in the nucleosome are referred to as super helical locations (SHLs). SHL 0 is the center of the nucleosomal DNA and each SHL locations, numbered from SHL −7 to +7, have ∼10 base-pair periodicities.

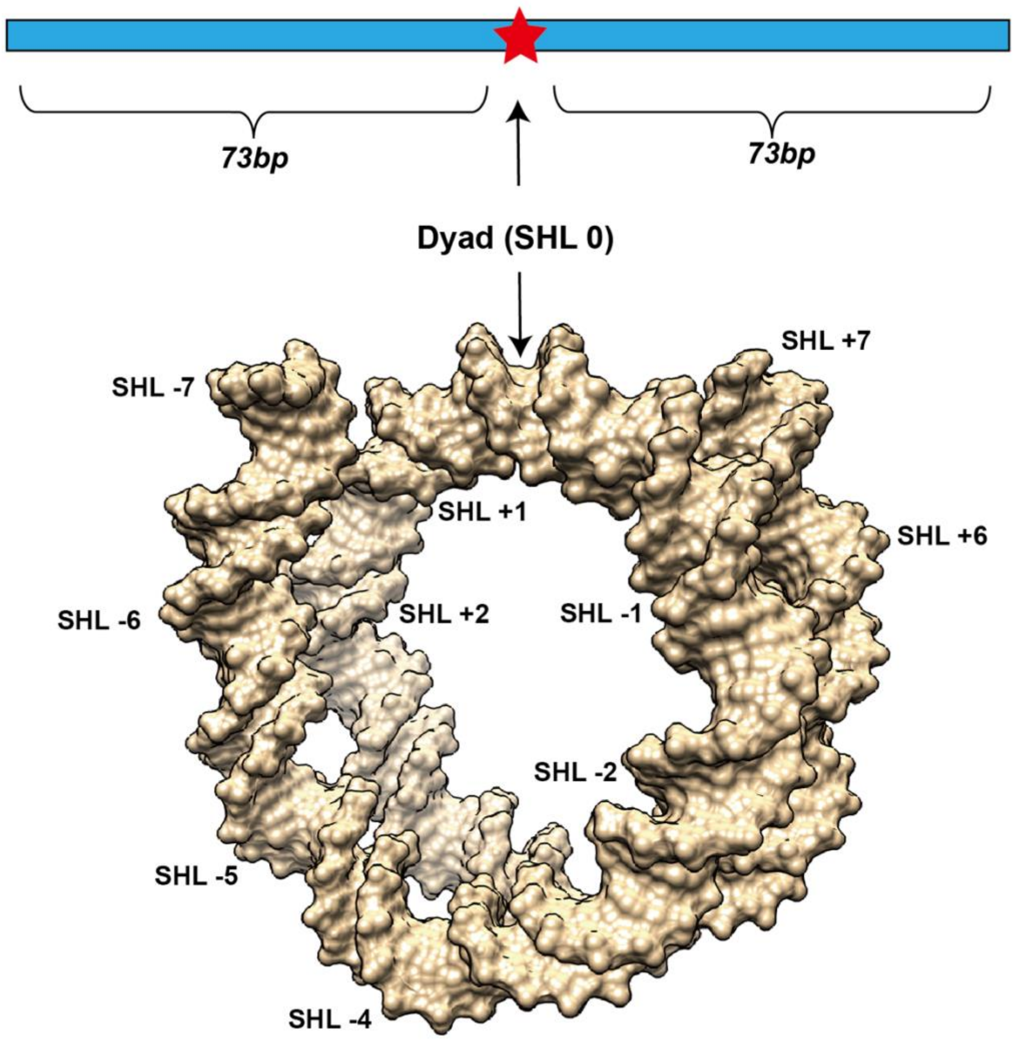

Reference

1 Steger, D. J. & Workman, J. L. Stable co-occupancy of transcription factors and histones at the HIV-1 enhancer. *EMBO J* **16**, 2463-2472, doi:10.1093/emboj/16.9.2463 (1997).

2 Qu, M., Qu, H., Jia, Z. & Kay, S. A. HNF4A defines tissue-specific circadian rhythms by beaconing BMAL1::CLOCK chromatin binding and shaping the rhythmic chromatin landscape. *Nat Commun* **12**, 6350, doi:10.1038/s41467-021-26567-3 (2021).

3 Bejjani, F., Evanno, E., Zibara, K., Piechaczyk, M. & Jariel-Encontre, I. The AP-1 transcriptional complex: Local switch or remote command? *Biochim Biophys Acta Rev Cancer* **1872**, 11-23, doi:10.1016/j.bbcan.2019.04.003 (2019).

4 Chaudhari, H. G. & Cohen, B. A. Local sequence features that influence AP-1 cis-regulatory activity. *Genome Res* **28**, 171-181, doi:10.1101/gr.226530.117 (2018).

5 Grebbin, B. M. & Schulte, D. PBX1 as Pioneer Factor: A Case Still Open. *Front Cell Dev Biol* **5**, 9, doi:10.3389/fcell.2017.00009 (2017).

6 Yang, Y. *et al.* Conversion of Fibroblast into Functional Leydig-like Cell Using Defined Small Molecules. *Stem Cell Reports* **15**, 408-423, doi:10.1016/j.stemcr.2020.07.002 (2020).

7 Onishi, Y. & Kiyama, R. Interaction of NF-E2 in the human beta-globin locus control region before chromatin remodeling. *J Biol Chem* **278**, 8163-8171, doi:10.1074/jbc.M209612200 (2003).

8 Shan, J., Fu, L., Balasubramanian, M. N., Anthony, T. & Kilberg, M. S. ATF4-dependent regulation of the JMJD3 gene during amino acid deprivation can be rescued in Atf4-deficient cells by inhibition of deacetylation. *J Biol Chem* **287**, 36393-36403, doi:10.1074/jbc.M112.399600 (2012).

9 Last, T. J., van Wijnen, A. J., de Ridder, M. C., Stein, G. S. & Stein, J. L. The homeodomain transcription factor CDP/cut interacts with the cell cycle regulatory element of histone H4 genes packaged into nucleosomes. *Mol Biol Rep* **26**, 185-194, doi:10.1023/a:1007058123699 (1999).

10 Ji, Z., Li, Y., Liu, S. X. & Sharrocks, A. D. Correction to article "The forkhead transcription factor FOXK2 premarks lineage-specific genes in human embryonic stem cells for activation during differentiation". *Nucleic Acids Res* **49**, 4198, doi:10.1093/nar/gkab221 (2021).

11 Zhu, F. *et al.* The interaction landscape between transcription factors and the nucleosome. *Nature* **562**, 76-81, doi:10.1038/s41586-018-0549-5 (2018).
